# A comprehensive survey of hearing questionnaires: how many are there, what do they measure, and how have they been validated?

**DOI:** 10.1186/1745-6215-16-S1-P26

**Published:** 2015-05-29

**Authors:** Michael A Akeroyd, Kay Wright-Whyte, Jack A Holman, William M Whitmer

**Affiliations:** 1 MCS/CSO Institute of Hearing Research – Scottish Section, Glasgow, G31 2ER, UK

## 

The self-report questionnaire is a popular tool for measuring outcomes in trials of interventions for hearing impairment. Many have been designed over the last fifty years, and there is no single standard questionnaire that is widely accepted and used. We felt it would be a valuable resource to have a comprehensive collection of all adult hearing-loss questionnaires (excluding those wholly devoted to tinnitus, children, or cochlear implants) and to survey their degree of validation. We collated copies of every published hearing difficulty questionnaire that we could find. The search was primarily done by iterative reference searching. Questionnaire topics were obtained by mapping the text of each questionnaire onto a set of categories; reports of validation methods were taken from the primary paper(s) on each questionnaire. In total we found 139 hearing-specific questionnaires (though many others were found that were primarily about something else). Though not formally systematic, we believe that we have included every questionnaire that is important, most of those of some notice, and a fair fraction of those obscure. We classified 111 as “primary” and the remaining 28 as “contractions”, being shortened versions of a primary without any new questions. In total, there were 3618 items across all the primary questionnaires. The median number of items per questionnaire was 20; the maximum was 158. Across all items, about one third were concerned with the person’s own hearing, another third with the repercussions of it, and about a quarter with hearing aids. There was a wide range in validation methods, from only using items chosen statistically from wider pools and with formal validation against independent measures of clinical outcomes, to just reporting a correlation with an audiogram measure of hearing loss. The “state of play” of the field of hearing questionnaires will be discussed.

